# Patient satisfaction and gender composition of physicians - a cross-sectional study of community health services in Hubei, China

**DOI:** 10.1186/s12913-018-3011-3

**Published:** 2018-03-27

**Authors:** Change Xiong, Xiao Chen, Xinyuan Zhao, Chaojie Liu

**Affiliations:** 1 Change Xiong, Department of Social Medicine and Health Management, School of Basic Medical Science, Hubei University of Science and Technology, 88 Xianning Road, Xianning, 437100 People’s Republic of China; 2 Xiao Chen, Institute of Medicine, Hubei University of Science and Technology, 88 Xianning Road, Xianning, 437100 People’s Republic of China; 3 Xinyuan Zhao, School of Clinic Medical Science, Hubei University of Science and Technology, Xianning, 437100 People’s Republic of China; 4 Chaojie Liu, School of Psychology and Public Health, La Trobe University, Melbourne, VIC 3086 Australia

**Keywords:** Patient satisfaction, Community health service, Gender composition

## Abstract

**Background:**

Patient satisfaction is associated with both individual (patients and health workers) and organizational (health facilities) characteristics. This study aimed to establish a link between patient satisfaction and gender composition of physicians in community health service (CHS) organizations.

**Methods:**

Participants were selected through multistage stratified random sampling: 36 CHS centers were selected from six municipalities in Hubei, China. All physicians on duty and patients visiting the CHS during the study period (from April to October in 2015) were invited to participate in this study: 324 physicians and 865 patients completed a questionnaire survey. Multilevel linear regression analyses were performed to determine the associations of patient satisfaction (scored from 1 to 5) with patient characteristics (gender, age, education, income, medical expense, frequency of visits to the CHS) and organizational features of the CHS (sex ratio of physicians, and gender differences of physicians in education and job satisfaction).

**Results:**

Older patients and those with a higher medical bill had a lower degree of satisfaction (*p* < 0.05). At the organizational level: a higher proportion of male physicians weakened the negative association between patient age and patient satisfaction (*p* < 0.05); a larger gap in university qualifications between male and female physicians exacerbated the negative association between patient age and patient satisfaction (*p* < 0.05).

**Conclusions:**

The gender composition of physicians in CHSs is associated with patient satisfaction in the Chinese context: a larger gap (in number and qualifications) between male and female physicians is associated with higher patient satisfaction. Further studies are needed to explore the cultural roots of such an association.

## Background

Patient satisfaction has been commonly used for evaluating health care services. It reflects the overall experience of a patient [1]. Patient satisfaction affects clinical outcomes, patient retention, and patient-doctor relationships [2]. Satisfied patients are more likely to comply with medical advice and be cooperative. In China, great efforts have been made to improve patient satisfaction with primary care [3], with an intention to divert patients from hospitals (often a very crowded environment) to community health services (CHSs) [4].

Patient satisfaction is shaped by at least four factors: the personal preference of the patient, the patient’s expectation, the response tendency of the patient due to personal characteristics, and the quality of care received [1]. Some researchers argue that patient satisfaction can also be influenced by the previous experience of the patient and the opinions of others, such as relatives and friends [5].

Extensive studies have been undertaken to identify the determinants of patient satisfaction [6–23]. A study in the USA found that older and literacy-deficient patients tend to have a higher degree of satisfaction than their younger and functionally literate peers [10]. Cohen in the UK also found that age is a strong predictor of patient ratings on satisfaction for both hospital and primary care, with older patients having a higher degree of satisfaction [8, 13]. This could be caused by the lower expectations of older patients [14, 18]. But some researchers argue that older patients are also likely to be treated with more respect by physicians [15]. The findings on gender differences in patient satisfaction are mixed, with some claiming that female patients have a higher degree of satisfaction and others reaching an opposite conclusion [13, 14].

Patient satisfaction can also be shaped by the characteristics and behaviors of health workers [8–10, 16]. Good communication and attitudes (such as understanding, listening, respect and empathy) can increase the degree of patient satisfaction [6, 17]. Compared with female physicians, male physicians tend to be more active and controlling in communications [18]. In some cultures, patients talk more, disclose more private information and make more positive statements to female physicians than to male physicians [19]. Harbinder [20] found that the gender of medical practitioners is associated with length of consultation, agendas elicited, and the content and style of communications. Cypress [21] reported that female doctors spend more time with female patients than with male patients. Studies in Western countries revealed that a female-sex role congruent communication style leads to a higher degree of patient satisfaction when women see a female physician [22, 23].

Patient satisfaction has been increasingly used in comparing the performance of healthcare organizations [24, 25]. Indeed, patient satisfaction is a reflection of the collective efforts made by a healthcare organization. Each patient is looked after by a team comprising physicians, nurses and other professional and administrative workers. Strong teamwork can improve the patient experience [26–28]. The strength of an organizational culture which puts patients at the center of care is positively associated with patient satisfaction [29]. Young and colleagues [30] found that larger and urban hospitals are usually rated lower by patients in satisfaction surveys compared with smaller and rural hospitals. Services involving graduate medical students may also lower patient satisfaction [31].

It is not clear whether the gender composition of the medical workforce, an important organizational feature, may influence patient satisfaction. Given the fact that gender differences exist among individual medical workers in responding to patient needs [32, 33], it is reasonable to expect an association between patient satisfaction and the gender composition of medical workers in an organization. However, it is important to note that the role of gender in different cultures may vary considerably.

This study was undertaken in China, with an aim to establish associations between patient satisfaction and the gender composition of physicians in CHS organizations.

We hypothesized that patient satisfaction is a multi-attribute construct, reflecting the experience of a patient over the process of interacting with a range of health workers and services in an organization. It is associated with the individual characteristics of the patient and the collective characteristics of the organization.

- Hypothesis 1: Patient satisfaction varies with the individual characteristics of patients, such as age, gender, education, and financial affordability of health care (measured by medical expenses and average household income);
- Hypothesis 2: Organizational characteristics as indicated by gender composition (in numbers, qualifications, and job satisfaction) in an organization moderates the effects of patient individual characteristics on patient satisfaction.

We did not assume a particular direction in the associations between patient satisfaction and organizational and patients’ individual characteristics because such associations often depend on the system environment in which services are delivered.

## Methods

### Setting

From 2009 to 2012, the number of male registered physicians employed by CHSs in China increased from 41,250 (46.8%) to 63,133 (48.1%), while female registered doctors increased from 46,947 (53.2%) to 78,750 (51.9%) [34]. Compared with developed nations such as Australia [35], Canada [36], the UK [37] and the USA [38], China has a higher proportion of female physicians working in primary care settings. Moreover, not all physicians in China hold a university medical degree. About 37% of physicians in CHSs completed a bachelor degree of medicine.

This study was conducted in Hubei province. Hubei is situated in central China, with a population of 58.5 million. It ranks in the middle range of economic development in all the provinces in China in terms of per capita GDP. There are 771 hospitals and 326 CHS centers, which employ about 65,800 and 5600 physicians, respectively [39].

### Sampling

A multistage stratified random sampling strategy was employed in this study. Seventeen municipalities in Hubei were divided into three economic zones according to per capita GDP. Two municipalities from each zone were randomly selected. Six CHS organizations were then randomly selected from each participating municipality. This resulted in a total sample of 36 CHS centers.

### Data collection

The number of male and female physicians was provided by the managers of the participating CHSs.

All physicians on duty and the patients visiting the participating CHS on the day when the survey was undertaken during the period from April to October of 2015 were invited to participate in a questionnaire survey. A total of 1319 questionnaires were dispatched: 387 to physicians and 932 to patients. Response rates were high, with 324 (83.7%) questionnaires being returned from the invited physicians and 865 (92.8%) questionnaires being returned from the invited patients. The questionnaire was administered through face-to-face interviews by six trained interviewers. Patients were interviewed after they had completed a visit to the CHS.

The questionnaire for physicians consisted of two parts. Part one collected information related to socio-demographic characteristics (e.g. age and sex) and professional training (e.g. qualification). Part two assessed job satisfaction. Previous studies found that the job satisfaction of medical workers is positively associated with patient satisfaction [40, 41]. The job satisfaction scale used in this study was adapted from the *Minnesota Satisfaction Questionnaire* (MSQ), which contains 12 items [42]. The MSQ has been validated and widely accepted internationally [43]. Example questions included how the respondents felt about the “decision-making ability of their superior” and their “job promotion opportunities”. Respondents were asked to rate their experiences on a five-point Likert scale [from very dissatisfied (score 1) to very satisfied (score 5)]. The item scores were added and averaged. The internal consistency of the MSQ scale in this study population is high, with an alpha coefficient of 0.839.

The questionnaire for patients also consisted of two parts. Part one collected information related to the socio-demographic characteristics of patients, such as age, gender, education, household income and frequency of visits to CHSs in the past year. Part two assessed patient satisfaction towards “quality of care”, “skills of physicians” and “attitudes of physicians”. Each of the three aspects was measured using one item: “Overall, how satisfied are you with the quality of care you received?” (quality of care); “How satisfied are you with the skills of your physicians who made the diagnoses and delivered treatment?” (skills of physicians); “How satisfied are you with the attitudes of your physicians?” (attitudes of physicians). Respondents (including 44 (5%) respondents on behalf of their < 18 years old children) were asked to rate items on a five-point Likert scale, ranging from 1 (strongly dissatisfied) to 5 (strongly satisfied). These items were chosen from the *Patient Satisfaction Questionnaire Short-form* (PSQ-18) [44] with consideration of their relevance and importance to CHS patients in China. Sixma and colleagues argue that patient expectations and concerns should be the utmost consideration in measuring patient satisfaction [45]. In general, patient expectations encompass a variety of dimensions, which range from the physical facility and convenience to skilled services and outcomes of care [3, 46, 47]. In response to patient expectations, health care services need to be delivered in a fair, compassionate and respectful way. Hall and Dornan [48] found that services that demonstrate humanness and competence, cost-effectiveness, good accessibility and share information are most valued by patients. Studies in China showed that the overwhelming concerns of CHS patients rest on quality of care, skill competency of physicians, and attitudes of health workers [49, 50].

### Outcome variable

Patient satisfaction was measured by the average score of the three items, which had an acceptable internal consistency (alpha =0.715).

### Independent variable

The gender composition of the medical workforce in CHSs is the major focus of this study. Three variables were generated to measure gender composition. The sex ratio (number of males to females) reflects the percentage distribution of gender in relation to physicians. We also measured differences in qualifications and job satisfaction between male and female physicians. We used the questionnaire for physicians to estimate the sex ratio in relation to the percentage of physicians with a university qualification. Gender differences in job satisfaction were measured by the difference in mean scores between male and female physicians.

A number of factors may have a confounding influence on the associations between patient satisfaction and the gender composition of physicians. In this study, we considered patient age, gender, education, average household monthly income, medical expenses relating to recent visits to a CHS, and the frequency of visits to a CHS over the past year.

### Data analysis

Multi-level linear regression analyses were performed using HLM7.0. We established a two-level random effect models using maximum likelihood estimation (MLE). The two levels of measurements corresponded to the individual characteristics of patients and the collective characteristics of CHSs, respectively (Table 1).

**Table 1:** Values assigned to the independent variables in multi-level modelling

Variable	Description and value
Individual-level (patients)	
Sex	0 = Male; 1 = Female
Age	Continuous variable measured as “years”
Education	0 = Without a tertiary qualification; 1 = With a tertiary qualification
Average monthly household income	Continuous variable measured in monetary terms “Yuan”
Medical expenses	Continuous variable measured in monetary terms “Yuan”
Frequency of visits	Continuous variable measured as “Times”
Organizational-level (community health services)	
Sex ratio “numbers”	Ratio of the number of male to female medical workers
Sex ratio “qualifications”	Percentage of male to female physicians with a bachelor degree
Gender difference in job satisfaction	Difference in mean scores of job satisfaction between male and female physicians

We started with an unconditional model (intercept only, Model One in Table 3) to examine the variations in the outcome variable (patient satisfaction) first.

We then developed a model (Model Two) to test hypothesis one, containing only level one variables (patient characteristics):

$$ {\mathrm{Y}}_{\mathrm{ij}}={\upbeta}_{0\mathrm{j}}+{\upbeta}_{1\mathrm{j}}\left(\mathrm{gender}\right)+{\upbeta}_{2\mathrm{j}}\left(\mathrm{age}\right)+{\upbeta}_{3\mathrm{j}}\left(\mathrm{education}\right)+{\upbeta}_{4\mathrm{j}}\left(\mathrm{income}\right)+{\upbeta}_{5\mathrm{j}}\left(\mathrm{medical}\ \mathrm{expense}\right)+{\upbeta}_{6\mathrm{j}}\left(\mathrm{frequency}\ \mathrm{of}\ \mathrm{visits}\ \mathrm{to}\ \mathrm{CHS}\right)+{\upgamma}_{\mathrm{ij}} $$

Level two variables (organizational characteristics) were subsequently introduced into the model to estimate the mixed effect regression coefficients. Because patient gender, education, household income and frequency of visits to CHS did not appear as a significant predictor of patient satisfaction, their mixed effects with level two variables were not explored. Model Three tested the interaction effect between “age” and level two variables, while Model Four tested the interaction effect between “medical expense” and level two variables (hypothesis two).

$$ {\upbeta}_{0\mathrm{j}}={\upgamma}_{00}+{\upmu}_{0\mathrm{j}} $$

$$ {\upbeta}_{1\mathrm{j}}={\upgamma}_{10}+{\upmu}_{1\mathrm{j}} $$

$$ {\upbeta}_{2\mathrm{j}}={\upgamma}_{20}+{\upgamma}_{21}\left(\mathrm{sex}\ {\mathrm{ratio}}^{``}{\mathrm{numbers}}^{"}\right)+{\upgamma}_{22}\ \left(\mathrm{sex}\ {\mathrm{ratio}}^{``}{\mathrm{qualifications}}^{"}\right)+{\upgamma}_{23}\ \left(\mathrm{gender}\ \mathrm{differences}\ \mathrm{in}\ \mathrm{job}\ \mathrm{satisfaction}\right)+{\upmu}_{2\mathrm{j}} $$

$$ {\upbeta}_{3\mathrm{j}}={\upgamma}_{30}+{\upmu}_{3\mathrm{j}} $$

$$ {\upbeta}_{4\mathrm{j}}={\upgamma}_{40}+{\upmu}_{4\mathrm{j}} $$

$$ {\upbeta}_{5\mathrm{j}}={\upgamma}_{50}+{\upgamma}_{51}\left(\mathrm{sex}\ {\mathrm{ratio}}^{``}{\mathrm{numbers}}^{"}\right)+{\upgamma}_{52}\left(\mathrm{sex}\ {\mathrm{ratio}}^{``}{\mathrm{qualifications}}^{"}\right)+{\upgamma}_{53}\left(\mathrm{gender}\ \mathrm{differences}\ \mathrm{in}\ \mathrm{job}\ \mathrm{satisfaction}\right)+{\upmu}_{5\mathrm{j}} $$

$$ {\upbeta}_{6\mathrm{j}}={\upgamma}_{60}+{\upmu}_{6\mathrm{j}} $$

Y_ij_ represents the satisfaction rating of patient i (level 1) in CHS j (level 2). βis a fixed parameter to be estimated.γis a CHS-specific random effect. Μis the component of error term.

## Results

### Characteristics of participants

Patient respondents (*n* = 865) had an average age of 44.65 (SD = 19.54) years: about 61% were female; and 22% had a university degree. Their average monthly household income was 3785 (SD 3466) Yuan RMB. On average, each patient visited a CHS five times in the past year and the most recent visit costed 464 Yuan RMB (Table 2).

**Table 2:** Characteristics of patient respondents and participating community health services (CHS)

Variable	Percentage	Mean	S.D.	Min.	Max.
Individual-level (patients)					
Proportion of female respondents	61%				
Age (Years)		44.65	19.54	1	96
Percentage of respondents with a university degree	22%				
Average monthly household income (Yuan)		3785	3466	100	50,000
Medical expense (Yuan)		464	898	2	8700
Frequency of visits to CHS		5.07	8.11	1	99
Organizational-level (community health services)					
Sex ratio (Male to Female) - numbers of physicians		0.65	1.14	0	7.00
Sex ratio (Male to Female) - percentage of physicians with a university degree		1.50	1.16	0	6.00
Gender difference in job satisfaction scores		0.7	4.83	−7.67	12.40

Physician respondents (*n* = 324) had an average age of 38.24 (SD = 10.92) years: about 54% were female; and 42% held a middle or senior professional title. On average, they had 16.25 (SD = 12.50) years of work experience.

The 36 participating CHSs had an average sex ratio of 0.65. Male physicians were more likely to hold a bachelor degree than their female colleagues (male to female ratio: 1.50). Male physicians had a significantly higher degree of job satisfaction than their female colleagues (*t* = 4.44, *p* = 0.000).

### Patient satisfaction and multilevel modelling

On average, patients gave a satisfaction score of 1.97 (SD = 0.65) to CHSs out of a maximum possible score of five. The unconditional model (Model One) revealed statistically significant variability in the outcome variable (patient satisfaction) across CHSs (*p* < 0.001, Table 3). The subsequent multilevel models indicated that heterogeneity in patient satisfaction scores existed across CHS organizations as measured by intraclass correlation coefficients (ICC). The ICC values range from 0 to 1. A non-zero ICC value is an indication of heterogeneity. The ICC values showed that organizational (level two) effects contributed to about 5.77% of the total variance in patient satisfaction. A reliability of 0.543 for Model One means that each sample of CHS is a reliable estimate of the population mean. Thus, a continued multi-level analysis is appropriate.

**Table 3:** Determinants of patient satisfaction scores - multilevel linear regression analyses

Independent variables	Model One		Model Two		Model Three		Model Four	
	Coef.	SE	Coef.	SE	Coef.	SE	Coef.	SE
Fixed effects								
Intercept (β_0_)			1.970	0.035	1.970	0.035	1.970	0.034
Individual-level (patients)								
Sex (β_1_)			−0.031	0.041	− 0.024	0.040	− 0.031	0.041
Age (β_2_)			− 0.005**	0.001	− 0.009**	− 0.002	− 0.005**	0.001
Education (β_3_)			−0.025	0.061	−0.033	0.060	−0.029	0.061
Monthly household income (β_4_)			0.000	0.000	0.000	0.000	0.000	0.000
Medical expenses for recent visit to CHS (β_5_)			−0.000*	0.000	−0.000	0.000	−0.000	0.000
Frequency of visits to CHS (β_6_)			−0.000	0.004	−0.000	0.004	−0.001	0.004
Organizational-level (community health services)								
Sex ratio (Male to Female) - numbers of physicians (β_i1_)					0.012*	0.005	0.001	0.000
Sex ratio (Male to Female) - percentage of physicians with a university degree (β_i2_)					−0.001*	0.000	−0.000	0.000
Gender difference in job satisfaction scores (β_i3_)					0.000	0.000	−0.000	0.000
Random effects								
Variance component		.0243 (μ_0_)		.0235		.0237 (μ_2_)		.0236 (μ_5_)
χ^2^(df)		97.688		90.604		91.800		91.212
*p*		< 0.001		< 0.001		< 0.001		< 0.001

Note: * *p* < 0.05; ** *p* < 0.01

In the regression model containing level one variables only (Model Two in Table 3), patient age and medical expenses relating to a recent visit to a CHS appeared to be significant predictors of patient satisfaction. Older patients and those with a higher medical bill had a lower degree of satisfaction (*p* < 0.05).

When level two variables were incorporated into the regression model for their interaction effects with level one variables “age” (Model Three) and “medical expense” (Model Four), the effect sizes of level one variables changed significantly (Table 3). The two-level mixed effect models showed that the negative association between age and patient satisfaction varied with the gender composition of physicians in CHSs; but the negative association between medical expenses and patient satisfaction did not vary with the gender composition of physicians in CHSs.

The organizational difference in the gender composition of physicians contributed to 5.89% of the total variance in patient satisfaction scores (which was estimated using intercept variance component of 0.0237 divided by intercept variance component plus level one variance component 0.379 + 0.0237). A higher percentage of male physicians weakened the negative effect of patient age on patient satisfaction. By contrast, a higher level of gender difference in qualifications exacerbated the negative effect of patient age on patient satisfaction.

## Discussion

### Patient satisfaction is associated with patient age and medical expenditure

This study revealed an overall low level of patient satisfaction with CHSs. In China, both hospitals and CHSs provide non-referred primary care to patients. It is not uncommon for patients to give lower ratings on CHSs in comparison with hospitals [11]. In addition, we found that older patients have a lower degree of satisfaction with CHSs than younger patients, which is different from the findings of some other studies [8, 9, 12–15]. Mitchell [9] argued that older patients are more enthusiastic about interacting with their physicians and therefore are more likely to be satisfied because their physicians are more likely to adopt a patient-centered approach with them. It is too early to jump to a conclusion about the reasons behind the lower degree of satisfaction of older patients in China. However, empirical studies show that patients of different ages prioritize different service dimensions in their satisfaction ratings. Older patients tend to value continuity more than younger patients do. In China, a shortage in the medical workforce is serious, which leads to heavy workloads for physicians and short consultations with patients [51]. Older patients often suffer from multiple and complex conditions. The inability of physicians to meet the demands of older patients for consultation time can translate into patient dissatisfaction [52, 53]. A mismatch between the needs of managing difficult physical and mental conditions of older patients and the training and professional skills obtained by primary care workers adds further complications to the situation [54, 55]. Physicians in primary care settings in China are often poorly equipped to confront the challenge [11].

Medical expenses were found to be negatively correlated with patient satisfaction, which is consistent with earlier studies [48, 56]. In China, out-of-pocket payments still impose a great financial challenge to patients, despite the rapid expansion of health insurance coverage. A recent study showed that low reimbursement for tuberculosis (TB) treatments in China leads to heavy financial burdens on patients and their families. The new rural cooperative medical scheme has limited effects on alleviating the financial burdens of its enrollees and improving equity [57]. Adding to the complexity is the fact that insured patients tend to stay longer in hospital and receive more treatments than those who are uninsured [58]. Vincenzo [59] found that out-of–pocket expenses in China decreased only for high income individuals with good health status and monetary savings increased only for low income individuals with good health status.

### Gender composition of physicians is linked to patient satisfaction ratings

We found that the gender composition of physicians in CHSs is associated with patient satisfaction in two ways. First, a higher percentage of male physicians weakens the negative association between patient age and patient satisfaction. Second, the gap in educational attainments between male and female physicians enhances the negative association of patient satisfaction with age.

A comparison of these findings with the results of other studies needs to be cautious. Previous studies found that female physicians tend to show more empathy through their non-verbal communications (i.e. smile and nodding), are more polite, and provide more advice to their patients than their male colleagues [20, 60–62]. However, this study did not link patient satisfaction with individual physicians. Gender composition was treated as an organizational feature.

The Chinese context may offer an explanation for our findings. Ting and colleagues [53] found that Chinese patients prefer an authoritarian approach rather than a more participatory approach in clinical decisions. They want health workers to act on their behalf without necessarily sharing the power in decision making. Both male and female patients prefer to choose a male doctor for consultations because they appear to have more “authority” than their female colleagues (a higher proportion of male physicians offers a better chance for such a choice) [63, 64]. This belief is well aligned with the hierarchical Chinese culture where men play a dominant role in society. Professional work is usually considered to be the responsibility of men while women stay at home to look after the family. Although women were encouraged to enter the workforce when Chairman Mao seized power in 1949 and saw women as “holding up half of the sky”, the stereotypical attitudes toward gender roles persist due to the thousands of years of influence of the Confucius philosophy. Interestingly, a study in California in the USA also demonstrated a higher degree of patient satisfaction with male doctors [65].

Male physicians are more likely to hold a university degree than female physicians in China. Understandably, patients tend to show more respect and satisfaction to those with a higher level of medical training. In general, CHS physicians in China are poorly trained. Many were transformed from “barefoot doctors” with limited vocational training [66]. Although university degree-based training for general practitioners started to attract attention in the 1980s [67], the current capacity of the university educational system is still very limited. For older patients with complex conditions, CHS physicians without a university degree can hardly meet their service needs [68].

## Conclusion

A low level of patient satisfaction with CHSs is evident in the study setting. Women comprise more than half the physicians in CHSs in China. The negative link between patient satisfaction and the proportion of female physicians in CHSs is concerning. A significant gap in university qualifications between male and female physicians exists, which exacerbates the negative association between patient age and satisfaction ratings. Strengthening the professional training of female physicians may contribute to the improvement of patient satisfaction, especially for older patients.

It is important to note that patient satisfaction with CHSs is associated with both the individual characteristics of patients (such as age and medical expenses) and the collective characteristics of organizations (such as the gender composition of physicians). Comparisons of CHS organizations based on patient satisfaction data should consider risk adjustments for differences in physician and patient profiles. Furthermore, managers should be encouraged to put more effort into enhancing the professional training of female physicians, rather than developing policies that discriminate against female physicians [69, 70].

### Limitation

This study was undertaken in 36 CHS organizations in Hubei province. The gender composition indicators are restricted to physicians in CHSs. The results should not be generalized to the entire hospital sector, nor to other healthcare workforce (such as nurses, pharmacists, allied health practitioners, and managers).

We have not been able to include patient diagnoses in the multi-level modelling due to unavailability of data. Further studies should consider patient casemix adjustment, if possible.

We established multi-level models using a summed patient satisfaction score based on three items because it is unreliable to transform a five-point Likert scale on a single item into a continuous measurement. We did not use the full scale of the PSQ-18 for concerns of overburdening the respondents. The β coefficients are small although they are statistically significant.

## Abbreviations

CHS: Community health service
GDP: Gross domestic product
ICC: Intraclass correlation coefficients
MLE: Maximum likelihood estimation
MSQ: Minnesota Satisfaction Questionnaire
PSQ-18: Patient satisfaction questionnaire short-form
TB: Tuberculosis
